# Effectiveness of Kinesiotherapy in the Treatment of Achilles Tendinopathy—A Narrative Review

**DOI:** 10.3390/sports12080202

**Published:** 2024-07-25

**Authors:** Robert Trybulski, Jarosław Muracki, Mieszko Podleśny, Andriy Vovkanych, Adrian Kużdżał

**Affiliations:** 1 Provita Żory Medical Center, 44-240 Żory, Poland; rtrybulski.provita@gmail.com; 2 Medical Department Wojciech Korfanty, Upper Silesian Academy in Katowice, 40-659 Katowice, Poland; 3 Institute of Physical Culture Sciences, Department of Physical Culture and Health, University of Szczecin, 70-453 Szczecin, Poland; 4 Salus Health Center, Zielona Street 8, 76-200 Słupsk, Poland; 5 Department of Physical Therapy and Ergotherapy, Ivan Boberkyj Lviv State University of Physical Culture, 79007 Lviv, Ukraine; 6 Institute of Health Sciences, College of Medical Sciences, University of Rzeszów, 35-310 Rzeszów, Poland

**Keywords:** tendons, non-invasive treatment, physical therapy, exercises with load, isometric loads, eccentric exercises

## Abstract

This narrative review of kinesiotherapy methods in the treatment of Achilles tendinopathy (AT) encompassed a diverse range of studies, including athletes and untrained people, healthy or injured, undergoing kinesiotherapy treatments. Most experimental studies (86%) reported results related to pain perception, 27% to the range of motion, and 27% to biomechanical assessment. However, the studies showed notable heterogeneity in the outcomes associated with the interventions, and, in this review of kinesiotherapy protocols for AT, a prominent observation emerged regarding their efficacy, suggesting a more favorable impact on pain and tendon stiffness management when comparing the measured parameters between the trained and untrained groups. The importance of tailoring the treatment approach based on the individual’s athletic background and conditioning status is underscored. There is a need for personalized rehabilitation strategies in athletic populations. The average duration of kinesiotherapy in the treatment of tendinopathy was 15.3 weeks. This observation underscores the potential of kinesiotherapy interventions as a viable treatment option for individuals with Achilles tendon issues. These findings underscore the urgent need for further research to provide stakeholders with more comprehensive directions for future studies. The results may be helpful for doctors, physiotherapists, trainers, and researchers interested in this topic.

## 1. Introduction

Tendon pathologies affect rising numbers of people in different countries who regularly exercise and who are predisposed to musculoskeletal disorders [1]. Tendinopathies continue to be a severe problem for the medical and sports community. The diseases included in the group of tendinopathies according to the primary classification are represented by tendonitis, tendinosis, paratenonitis, and paratenonitis with tendinosis, which, unfortunately, are still unevaluated epidemiologically [2]. In the current scientific literature, one can find statistical and epidemiological data on a concrete nosological unit of tendinopathies for a particular sport [3]. Still, no systematic analyses of all types of tendinopathies are presented.

It is even more difficult to find statistics on patients who do not play sports professionally. However, D. Challoumas et al. published scientific data showing that injuries of the tendons, ligaments, and joint complexes of the musculoskeletal system in people who do not play sports can account for up to 40% of all musculoskeletal injuries [4]. According to S. L. Hanlon et al., about 30% of tendinopathies occur in the knee joint area, and 60% of them involve the extensor apparatus of the joint, i.e., the ligaments of the patella and quadriceps femoris [5]. There are also currently many plans and clinical guidelines for the treatment and management of patients with tendinopathies, but there are no adequate evidence-based studies.

Achilles tendinopathy is defined as persistent pain in the Achilles tendon and loss of function related to mechanical loading [6]. According to K. Merry et al., Achilles tendinopathy can be divided depending on where the pathology occurs: (1) insertion tendon—symptoms located 0–2 cm from the distal attachment (20–25% of Achilles tendon injuries); (2) middle tendon—symptoms situated 2–7 cm from the insertion (55–65% of Achilles tendon injuries) [7].

The clinical features of tendinopathy diagnosis, as outlined by ICON 2019 [6], emphasize the significance of biomechanical physical tests conducted by a specialist, particularly in asymptomatic tendinopathy. As per W. Matthews et al., alongside comprehensive anamnestic data collection, it is crucial to pursue supplementary instrumental studies, notably an ultrasound examination of the joint and ligamentous apparatus [8]. This modality proves highly informative during the physiotherapy diagnosis phase, aiding in identifying tendon abnormalities and providing valuable insights into the patient’s condition. Through meticulous assessment utilizing biomechanical tests and advanced imaging techniques like ultrasound, clinicians can effectively pinpoint the tendinopathy, enabling tailored treatment strategies to address the patient’s specific needs and optimize the rehabilitation outcomes.

However, considering modern molecular research, the issue of understanding the pathogenesis of tendinopathies remains crucial in choosing a treatment strategy and in patient management. Unfortunately, there is no single theory in this area, but, based on recent research, the authors of this analysis identify three primary reaction cascades underlying tendinopathies [9]. One of them is a complex of responses of “failed healing” [10], which occurs despite the formation of new blood vessels. Alterations happening at different stages of tendon healing can lead to various manifestations of histopathological changes. Scientists describe the so-called “vicious circle” of tendinopathy pathogenesis as an interaction involving the susceptibility of the healing process to opposing traumatic mechanical, biochemical, and environmental factors, including tobacco smoking [11]. Another systematic analysis of current data on the pathogenesis of tendinopathies, conducted by Jaworski et al., suggests that, during the development of tendinopathies, the process of the disordered restructuring of the extracellular matrix of connective tissue occurs. This process is accompanied by the increased activity of enzymes and inflammatory factors [12]. The authors argue that, in many cases, the histological examination of the tendon tissue of the biopsy material is crucial in establishing the clinical diagnosis and the degree of tendon degeneration. Composed primarily of type I collagen fibers aligned in parallel, the tendon exhibits remarkable tensile strength and stiffness, capable of withstanding forces exceeding the body’s weight during dynamic activities. Its viscoelastic nature allows it to absorb and release energy efficiently, contributing to the energy-saving mechanism of locomotion [13]. However, its relatively poor blood supply and limited capacity for self-repair make it susceptible to injury, particularly during sudden, high-intensity movements or repetitive stress, underscoring the importance of proper conditioning and rehabilitation strategies to maintain tendon health and function.

Scientists have proven that the impaired tightness of the vascular endothelium in tendinopathies can be the main factor in the development of a pathogenic “vicious circle” and tendon degeneration. Such vessels are considered defective and cannot take part in the blood supply to the connective tissue of the tendon. Recent discoveries in vascular biology have introduced a range of genes responsible for stabilizing vasculogenesis in developing such pathologies. An analysis of these genes, published by K. I. Takino et al., showed that the R-Ras-Akt axis causes endothelial lumenogenesis and regulates the patency of regenerating vessels during pathogenic changes [14]. In the context of these reports and preliminary data on long-term tendon oxygen deficiency driven by genetic factors of endothelial modification, it is worth paying attention to kinesiotherapy methods and their evolution, as they show reliably effective results without invasive techniques.

Therefore, this study aimed to evaluate the current literature on evidence-based training and kinesiotherapy methods in the treatment protocol for Achilles tendinopathy in adult patients without injectable or surgical procedures and comment on their effectiveness.

## 2. Materials and Methods

The eligibility criteria for this narrative literature review were designed to be inclusive, focusing on individuals diagnosed with Achilles tendinopathy. Both acute and chronic cases were considered, without any limitations based on age, gender, or other demographic factors. This approach ensured the comprehensive representation of the affected population, making the findings of this review relevant to a wide range of readers. The intervention criteria focused on kinesiotherapy used to treat Achilles tendinopathy, encompassing a range of therapeutic exercises and manual techniques. These interventions aimed to relieve symptoms, improve function, and promote tendon healing. The study results were defined to include the most important measures related to the effectiveness of kinesiotherapy in the treatment of Achilles tendinopathy, such as pain levels, functional improvement, biomechanical parameters, patient-reported outcomes, and other relevant indicators of treatment effectiveness and patient satisfaction. A comprehensive full-text review was conducted to ensure methodological accuracy and relevance to the research question, thus providing valuable information on the effectiveness of kinesiotherapy interventions in the treatment of Achilles tendinopathy.

A meticulous strategy was employed to identify relevant studies, which involved searching three critical databases, PubMed, Scopus, and Web of Science, up until 22 March 2024. This comprehensive search approach ensured that the review encompassed the most up-to-date and relevant studies, enhancing the reliability and comprehensiveness of the findings. The researchers reviewed the searched literature, assessing each study’s eligibility for inclusion based on predefined criteria.

Details regarding the intervention settings were documented, including the study design, duration, training context, and specific aspects of the kinesiotherapy intervention. During data extraction, the main focus was on parameters related to acute reactions, including pain, fatigue, recovery, physical readiness, and sports performance.

The data synthesis process included a narrative synthesis supplemented with data summaries to provide a robust overview of the study findings.

## 3. Results and Discussion

### 3.1. Clinical Assessment of Achilles Tendinopathy

Achilles tendinopathy is an acute injury with severe pain from physical overuse, which is most common among professional athletes, specifically those involved in active running and jumping [15]. Apart from pain, Achilles tendinopathy is accompanied by structural and mechanical changes in the tendon, which leads to the impaired function of the affected lower limb in general and sometimes the development of a psycho-emotional disorder in the form of a fear of movement [16,17]. Kinesiotherapy of Achilles tendinopathies can include concentric, eccentric, and isometric exercises, often used in rehabilitation protocols and the treatment of Achilles injuries. According to the Consensus of the International Scientific Symposium on Tendinopathy 2020 [18,19], tendinopathy symptoms are not limited to pain and limited limb mobility. They are also associated with the patient’s ability to work, their quality of life, depressive disorders (especially in professional athletes), and anxiety.

Achilles tendinopathy is the most frequently examined tendon injury using ultrasonic elastography (USE), especially in the postoperative period, after the surgical repair of a ruptured Achilles tendon and as an assessment of “asymptomatic” tendons [20]. The early diagnosis of tendinopathy is crucial to implement conservative measures to avoid serious tendon injury or even rupture [21,22]. Concerning conventional ultrasound (US), USE potentially increases tendinopathy’s sensitivity and diagnostic accuracy and can detect pathological changes before they become visible in conventional US imaging [20,22]. Compression elastography (CE) and shear wave elastography (SWE) are mainly used in the literature [20,21]. Studies confirm the high specificity and repeatability of this method [23,24].

Elastography allows one to detect areas of changed elasticity, which may indicate micro-injuries, inflammation, or tissue degeneration [21]. This may help to distinguish chronic inflammation from an acute injury, which is essential when planning physiotherapy treatments [20]. Regular elastography tests enable the monitoring of changes in tendon elasticity, which allows one to assess the effectiveness of the therapy and adjust the intensity and type of exercises [22]. Elastography provides data that can be used in research on the biomechanics of the Achilles tendon and its adaptation to various loads and pathological processes [25]. The limitations of this method are the need to have reference values, difficulties in interpreting the results, the need to have high-quality but expensive equipment, and the high level of diagnostic skill required of the examiner. There are no standardized testing protocols [20]. Therefore, this is not a standard method, such as myotonometry [26,27].

Measurements of the stiffness and elasticity of the Achilles tendon using a myotonometer are becoming very popular in clinical practice [27,28]. Myoton is a digital device with a body and a depth probe (Ø 3 mm). Initial pressure (0.18 N) is applied to the surface through the probe, which compresses the material underneath. The device then triggers a mechanical pulse (0.4 N, 15 ms), removing the medium quickly [29]. Myotonometry is a reliable measurement method and allows one to detect differences in physical properties compared to stretched muscle fibers [28]. The measurement method involves recording the damped natural vibrations of soft biological tissue in the form of an acceleration signal and then simultaneously calculating the parameters of the stress state and biomechanical properties [26]. Thanks to the device, one can assess, among other aspects, the state of resting muscle tension, defined as a muted EMG signal and dynamic stiffness. Stiffness assessment by symmetry is based on the theory of free oscillations and results from natural tissue oscillations in response to short-term mechanical exposure to the skin [29]. Tissue can also regain its original shape after deformation. This property, measured in this test, is called elasticity. The greater the elasticity, the faster the tissue returns to its original shape [27].

For the initial preliminary diagnosis of Achilles tendinopathy, three basic approaches are used: the palpation of the affected lower extremity, the assessment of pain, and the Royal London Hospital test, as described in N. Maffulli et al. [30]. The application of these approaches is presented in Table 1. Apart from the proposed methods, some authors suggest using the TENDINS-A (TENDINopathy Severity Assessment Achilles) questionnaire [31]. All of these methods have one common limitation—they are based on palpation and the subjective feelings of the patient and do not provide insights into the quality of the tendon’s tissue.

### 3.2. Application of Various Kinesiotherapy Regimens for the Treatment and Rehabilitation of Adult Patients with Achilles Tendinopathy

A clinical study by M. Gatz et al. included two experimental groups of patients with a clinically confirmed diagnosis of Achilles tendinopathy, with 20 and 22 patients in each group, respectively. The study aimed to compare the effectiveness of isometric and eccentric exercises in tendon rehabilitation; the first group performed eccentric exercises only, while the second group performed a combination of eccentric and isometric exercises, for three months, with a clinical assessment at the end of the first and third months. The Victorian Institute for Sports Assessment—Achilles (VISA-A) scale, the American Orthopedic Foot & Ankle Society scale, and shear wave EG were used to assess the tissue condition. In both groups, there was a considerable improvement in tendon function, but no significant individual differences were found in either group. The symptomatic insertion and central medial Achilles tendon showed a significantly lower Young’s modulus than asymptomatic, healthy tendons. Thus, isometric exercise has no proven benefits in treating tendinopathies in combination with eccentric exercises, as demonstrated by the results of a three-month controlled follow-up [32].

D. A. Prudêncio et al., in a meta-analysis of eight studies, showed that existing evidence favors the utilization of eccentric exercise for Achilles tendinopathy management. A sustained load on the Achilles tendon does not negatively impact pain and function outcomes, hinting at the feasibility of engaging in certain physical activities during treatment [33]. While some studies suggest comparable outcomes between eccentric and heavy slow resistance exercises, further research is necessary for confirmation. Additionally, rest/wait-and-see approaches likely do not contribute significantly to AT management, as indicated by some authors. N. A. Abdelkader et al. compared the effectiveness of an eccentric exercise program on the calf muscle group, followed by stretching and elasticity exercises. All patients were clinically diagnosed with chronic Achilles tendinopathy. Clinical follow-up was conducted over two years to assess the short- and long-term results. Notably, in contrast to the previous study by M. Gatz et al., N. A. Abdelkader included patients with unilateral tendinopathy for whom treatment with NSAIDs and steroids was not effective, as well as patients with concomitant pathologies of the musculoskeletal system (osteoarthritis, radiculopathy, systemic neurological pathologies) in the clinical group. The authors also used the VISA-A scale and the visual analog scale to assess the condition of the tendon. The Achilles tendon function and pain scores in the clinical group did not differ significantly from those in the control group during the first few months. However, they improved significantly after treatment in both study groups. After one and a half years of treatment, the results slightly but significantly decreased in the clinical group and increased in the control group of patients, and they were statistically favorable for symptomatic indicators compared to baseline. At both time points, the study group performed significantly better (statistically and clinically) than the control group [34].

To improve the elasticity of the Achilles tendon, A. Cini et al. investigated and showed the effectiveness of passive, static stretching exercises regardless of the number and duration of physical activity sessions, but with at least 2 min per affected limb [35]. According to A. C. Van Der Vlist et al., passive observation without the prescription of physical rehabilitation activities is not recommended for tendinopathy of the middle section of the Achilles tendon, as active treatment methods with exercise show satisfactory results regarding the joint complex capacity in the long term, within three months of observation. Contrary to previous studies, the authors of this study conclude that there is no clinically significant difference in effectiveness between different kinesiotherapeutic treatments. The authors emphasize the importance of the early prescription of kinesiotherapy, considering the range of apparent advantages: the ease and accessibility of prescription, its low cost, its non-invasiveness, and its beneficial effect not only on the tendon tissue mass but also on the calf muscles and adjacent tissues [36]. The authors of the same scientific school, in their practical study involving 91 patients with chronic Achilles tendinopathy of the middle part of the tendon, examined physical loads with isometric exercises for the calf muscle group (on tiptoe, with backward flexion of the ankle joint) and isotonic exercises for the calf muscle group in three clinical observation groups, as well as rest (control group). The pain was measured using a visual analog scale during and after the physical activity. The study did not show an analgesic effect after patients with chronic tendinopathy of the middle section of the Achilles tendon performed isometric exercises [36]. These results correlate with the scientific position of K. J. von Rickenbach, who states that in almost a third of cases of Achilles tendinopathy, chronic recurrent symptoms can develop, which require the use of invasive treatments in combination with conservative ones to achieve positive results regarding the patient’s quality of life [37]. These methods include injection techniques using steroid and anti-inflammatory medicines, prolotherapy, and microsurgery. Such data are supported by the findings of a systematic analysis by M. Kim et al., who argue that eccentric exercise training should be the first choice in managing patients with Achilles tendinopathy to improve their strength performance [38].

In contrast to studies with eccentric exercise, the clinical trial by C. H. Yeh et al. focused on evaluating the effectiveness of heavy, slow resistance training for patients with mid-segment Achilles tendinopathy. With an analogous external load, the force wave passing through the patella was more significant in eccentric loading with the knee bent than during slow resistance loading with the knee straightened, which is explained by the increased angle of posterior flexion in the ankle joint. A statistical data analysis showed that external loading and the highest posterior flexion angle were statistically significant factors in the peak patellar force in both standing and sitting positions. The authors conclude that in order to improve the quality of physical activity on the anterior tibial joint during the rehabilitation of the Achilles tendon, an adequate (depending on the clinical case) external load should be prescribed and the maximum posterior flexion should be controlled during exercise. The findings also suggest that the peak angle of posterior flexion of the limb affects the strength of the Achilles tendon in a standing position significantly more than in a sitting position. Therefore, the standing position may be more favorable with a uniform external load and peak angle of posterior flexion. The authors suggest that the duration of symptoms may not correlate with the prognosis of mid-segment Achilles tendinopathy, and physical exercises, apart from exerting a strong influence on the dynamics of rehabilitation, have shown a favorable effect on the psycho-emotional states of patients during rehabilitation [39].

S. L. Hanlon et al. aimed to assess the effect of the time since the onset of Achilles tendinopathy and the leading indicators of tendon function on the results among patients with different durations of symptoms after four months of complex exercise treatment. The study’s authors divided 127 patients into groups depending on the time ranges from the onset of the first sympathetic manifestations of tendinopathy. They received 16 weeks of standardized physical therapy and activity modification tailored to their pain syndrome. To assess the effectiveness of the therapy, the strength of the lower extremities, the structure of the tendons, their mechanical properties, and the psychological impact on the patient’s well-being were evaluated. The indicators were analyzed before the study and at 2 and 4 months of follow-up. After four months of regular performance of the prescribed set of physical exercises during the rehabilitation of tendinopathy of the middle segment of the Achilles tendon, all observed groups of patients showed improvements in symptoms and in the functional ability of the lower extremities, without significant differences between the groups. An improvement in the tendon structure was noted by ultrasound diagnostics (US). The duration of tendinopathy symptoms did not statistically affect the disease’s clinical manifestation at the baseline tendon function assessment [40].

Data have been published on the effectiveness of high-load exercise in Achilles tendinopathy in terms of mechanical factors (tissue stiffness, maximum tendon extension), histological factors (cross-sectional area), the maximum isometric plantar flexor strength, rehabilitation adaptation (VISA-A score), and pain (numerical scale to assess the severity of pain) as the primary outcomes of therapy. As the secondary outcomes of treatment in a blind study, G. Radovanović et al. assessed the fall and jump heights against motion and ultrasound measures of branching and the topography of the intratendinous blood supply system. The researchers conducted a three-month follow-up of male patients with a clinically confirmed diagnosis of chronic Achilles tendinopathy. Depending on the clinical group, patients were prescribed high-impact exercises, eccentric exercises (according to the Alfredson protocol) as standard therapy, and passive static therapy. Despite the positive dynamics and improvement in tendon function, it was in the clinical group that performed high-impact physical activity that the development of mechanical and morphological adaptive reactions of the plantar flexor musculotendinous complex was noted. The authors explain this fact by the protection of the tendon structure from the damaging force of the traumatic wave caused by overstrain. The clinical trial authors recommend using high-load kinesiotherapy as an effective, non-invasive therapeutic method in the rehabilitation protocol for Achilles tendinopathy in men, regardless of the segment of the tendon lesion [41].

A clinical randomized trial conducted in Poland by M. Stania et al. included a three-month follow-up of patients with non-invasive Achilles tendinopathy and pain syndrome, who, in addition to standard treatment (including deep friction massage), were prescribed radial shock wave therapy, ultrasound therapy, or a placebo. The authors showed statistically significant results in terms of two-factor interactions between the type of therapy and physical activity. All three non-invasive treatment methods showed satisfactory results within the same statistical limits, so one method could not be singled out as a superior choice [42].

In the study of Corrigan et al., ultrasound diagnostics of the Achilles tendon were performed, the results of which can correlate with the prognosis of symptomatic manifestations. Thus, according to R. Corrigan, the considerable thickening of the tendon tissue on initial ultrasound is associated with more severe symptomatic manifestations. Lower viscosity at the initial examination of patients also correlated with the poorer endurance of the calf muscles in patients with tendinopathy [43].

### 3.3. Pain Component in the Rehabilitation of Achilles Tendinopathy and Treatment with Kinesiotherapy

Pain syndrome in Achilles tendinopathy causes considerable functional limitations in the movements of the lower limb, which reduces the patient’s quality of life and adds to the social burden [44,45]. S. Spang et al. showed that, in patients with chronic pain syndrome caused by patellar tendinopathy, especially with acute pain on the superficial side of the proximal patellar tendon, the sensory nerves could be topographically located not only on the dorsal side but also on the superficial surface of the tendon, which explained the patients’ severe clinical manifestations [46]. It is vital to investigate the psycho-emotional states of patients undergoing kinesiotherapeutic rehabilitation for chronic tendinopathy. As noted by H. Alfredson and J. Cook, the cause of pain in chronic tendinopathy may be neovascularization and nerve-ending proliferation, which occurs in response to a traumatic tendon injury [47]. According to A. Mallows et al., who conducted a clinical follow-up of patients undergoing exclusively physiotherapy rehabilitation in private medical centers for chronic tendinopathy of various localizations, it is essential to ensure a flexible, empathetic, supportive approach in the rehabilitation process, with an emphasis on the patient’s psychosocial state, for positive dynamics [48].

Non-surgical, non-invasive treatment with eccentric loads and training, according to H. Alfredson, is the first line of treatment for chronic tendinopathy with severe pain, especially in the clinical variant consisting of lesions in the middle section of the Achilles tendon. H. Alfredson’s study of innervation in patients with chronic tendinopathy of the medial Achilles tendon showed that, histologically, there were no nerve endings inside the affected medial part of the tendon. However, the pathological examination revealed nerve endings and nerves outside the tendon’s inner surface [49].

According to the findings of the literature review, summarized in Table 2, and other meta-analyses on the effectiveness of kinesiotherapy in the treatment of tendinopathies, the best results in the short and long term are shown with combinations of physical activity of different amplitudes and directions on the limb with the affected tendon. P. A. Swinton et al. reached an analogous conclusion, analyzing two hundred studies with more than 450 treatment areas [50]. The authors concluded that physicians should consider a combination of exercise and non-physical treatments as a fundamental approach in the evidence-based treatment of tendinopathy. According to the results of clinical observations over the past five years, the most effective treatment combinations include physical kinesiotherapeutic exercises using biomechanical therapy.

## 4. Limitations

The limitations of this narrative review include the insufficient number of studies, although they brought substantial and statistically significant results to the analysis. Our search included only seven studies in the analysis. Another limitation is the fact that researchers use different methodologies to assess the tissue characteristics and patients’ feelings, which makes it impossible for the studies to be compared directly. Another severe limitation is that the currently available research articles analyze different groups—athletes or untrained people of different ages—and at different times from tendinopathy onset, which makes the results challenging to interpret. All of these limitations can be treated as future research directions, aiming to conduct RCT studies with well-established methodologies.

## 5. Conclusions

The combination of eccentric physical activity in the calf muscle group with tendon stretching exercises has considerably improved the pain and functional performance of patients with chronic unilateral Achilles tendinopathy when used for at least six months of regular sessions. Eccentric training has shown positive, evidence-based results in clinical trials, able to improve the kinetic parameters of strength performance in patients with chronic tendinopathies, such as the height of the long jump. With the use of eccentric loading, it is possible to apply a higher mechanical load compared to concentric loading, as eccentric exercise can cause tendon overstretching and, as a result, lead to a negative mechanical load on the tendon.

The majority of the analyzed studies used a standard set of diagnostic methods, including VISA-A, ultrasound, algometry, a visual analog scale, and elastography. Combinations of various types of physical activity, such as isometric and isotonic exercises, or a set of static exercises with a heavy, slow load and resistance, showed the most effective indicators in terms of kinesiotherapy treatment. While evidence supporting the efficacy of kinesiotherapy in Achilles tendinopathy is growing, further research is needed to fully understand its optimal role in pain reduction and the overall management of the condition.

Practitioners and rehabilitators are recommended to prescribe a combination of physical exercises and non-physical methods of tendon rehabilitation as a fundamental approach in the evidence-based treatment of tendinopathy of varied localization and duration. Further research on this topic should focus on comparing the clinical results of kinesiotherapy after minimally invasive surgical methods that involve correcting Achilles tendon injuries that have led to chronic tendinopathy.

## Figures and Tables

**Table 1:** Diagnostic approaches for the initial diagnosis of Achilles tendinopathy.

No.	Methodology	Appropriateness of Use in the Initial Examination
1	Palpation of the affected area	The Achilles tendon area is palpated for tenderness, localized fever, compaction, nodulation, and crepitation.
2	Pain in mid-portion (located 2–6 cm from the calcaneal insertion)	Determining the location of the pain syndrome is a vital criterion for the differential diagnosis between tendinopathy and para-tendinopathy. Often, a discrete node is identified, the intensity of which decreases or disappears when the tendon is loaded.
3	The Royal London Hospital test	An assessment of local tenderness during the palpation of the affected area when the ankle joint is inactive. Pain is considerably reduced when the ankle joint is flexed backward.

**Table 2:** Comparative characteristics of the leading clinical observations analyzed in the study.

No.	Authors and Year of Publication of Results	Number of Patients (Total)	Methods of Treatment and Rehabilitation Tested by Authors	Technique That Demonstrated Positive Effect on Tendinopathy in Statistically Significant Way	Methods of Clinical Assessment of Achilles Tendon Function and Condition
1	M. Gatz, M. Betsch, T. Dirrichs, S. Schrading, M. Tingart, R. Michalik, V. Quack, 2020 [32]	42	Various combinations of isometric and eccentric exercises	Both methods showed equivalent results (isometric and eccentric exercise)	VISA-A, American Orthopedic Foot and Ankle Society scale, EG of shear waves
2	N. S. B. Mansur, F. T. Matsunaga, O. L. Carrazzone, B. S. Dos Santos, C. G. Nunes et al., 2021 [51]	119	Non-kinesiotherapy approach and eccentric exercises	Set of physical exercises for at least three months	VISA-A, algometry, visual analog scale
3	N. A. Abdelkader, M. N. K. Helmy, N. A. Fayaz, E. S. Saweeres, 2021 [34]	50	Eccentric loading program followed by stretching exercises and a non-kinesiotherapy approach	Calf eccentric loading with stretching exercises for 3–4 months	VISA-A, visual analog scale
4	A. C. Van der Vlist, P. L. van Veldhoven, R. F. van Oosterom, J. A. Verhaar, R. J. de Vos, 2020 [36]	91	Different variations of isometric and isotonic exercises	Lack of analgesic effect and positive dynamics	visual analog scale
5	C. H. Yeh, J. D. Calder, J. Antflick, A. M. Bull, A. E. Kedgley, 2021 [39]	18	Complex static exercises with a heavy, slow resistance load	Complex static exercises with a heavy, slow resistance load	Vicon Motion Systems, kinematic analysis in software
6	S. L. Hanlon, R. Scattone Silva, B. J. Honick, K. G. Silbernagel, 2023 [40]	127	A standard set of physical exercises for four months	A standard set of physical exercises for at least two months	Ultrasound, questionnaire, physical examination, elastography
7	G. Radovanović, S. Bohm, K. K. Peper, A. Arampatzis, K. Legerlotz, 2022 [41]	39	Physical exercises with a high load	Physical exercises with a high load	VISA-A, numerical pain rating scale, ultrasound, morphological characteristics of tendon

## Data Availability

Not applicable.
